# Two Cases of Allergic Contact Dermatitis Caused by Coco Betaine in Clobetasol Propionate Shampoo

**DOI:** 10.1155/crdm/9953943

**Published:** 2026-01-02

**Authors:** Shigeruko Iijima, Kayo Murayama, Noriko Takayama, Mariko Sugiyama, Kayoko Matsunaga

**Affiliations:** ^1^ Division of Dermatology, Hanamizuki Clinic, Ushiku, Ibaraki, Japan; ^2^ General Incorporated Association SSCI-Net, Nagoya, Aichi, Japan; ^3^ Fujita Health University, Toyoake, Aichi, Japan, fujita-hu.ac.jp; ^4^ Department of Dermatology and Allergy, Kariya Orthopedic Hospital, Kariya, Aichi, Japan

**Keywords:** allergic contact dermatitis, amphoteric surfactant, CAS RN 61789-40-0, CAS RN 68424-94-2, clobetasol propionate shampoo, cocamidopropyl betaine, coco betaine

## Abstract

We present two cases of allergic contact dermatitis caused by the amphoteric surfactant coco betaine, a constituent of clobetasol propionate (CP) shampoo, despite the low allergenic potential of CP shampoo formulations. The patients were Japanese males, aged 49 and 52 years, with severe atopic dermatitis, who had been treated with oral cyclosporine for several years. Patch testing revealed that only coco betaine 1% aq. showed a clearly positive reaction in both cases, whereas the patch test results of CP shampoo, cocamidopropyl betaine, and lauramidopropyl betaine were positive in one case and doubtful in the other. In cases of recalcitrant scalp dermatitis, it is necessary to consider the possibility that it is being caused by the topical medication used to treat it, and that the “active” ingredient may not be the culprit.

## 1. Introduction

Clobetasol propionate (CP) shampoo (Comclo®; Maruho Co., Ltd., Osaka, Japan) contains a super high–potency topical corticosteroid and several surfactants. In Japan, CP shampoo was first approved as a treatment for scalp psoriasis in July 2017, and it was later approved as a treatment for scalp eczema in February 2021. Recently, Ramani et al. [1] reported that CP shampoo formulations contain fewer potential allergens than other CP‐containing products. In this report, we present two cases of allergic contact dermatitis caused by the amphoteric surfactant coco betaine, one of the additives of CP shampoo.

Our report is unique because only a few cases of allergic contact dermatitis triggered by additives, especially surfactants such as coco betaine, have been reported in the literature [2, 3], and patch tests of cocamidopropyl betaine (CAPB)/lauramidopropyl betaine (LAPB) were also performed in our cases, as they share some structural similarities with coco betaine.

## 2. Case Report

### 2.1. Case 1

**Figure 1:**
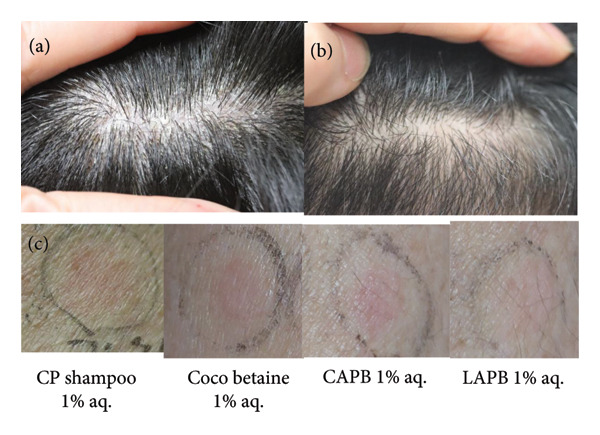
Clinical (a, b) and patch test findings obtained on D3 (c) in Case 1. Clinical findings of Case 1 before (a) and after (b) the use of CP shampoo was discontinued: (a) marked dandruff on the scalp and (b) a marked improvement. (c) Positive patch test results for CP shampoo 1% aq., coco betaine 1% aq., CAPB 1% aq., and LAPB 1% aq. on D3. CP: clobetasol propionate, CAPB: cocamidopropyl betaine, LAPB: lauramidopropyl betaine.

A 49‐year‐old Japanese male with scalp dermatitis was using betamethasone butyrate propionate (Antebate®) lotion. After a positive patch test of his previous shampoo, it was replaced with a less irritating one, resulting in his scalp being in good condition for 1.5 years. However, significant dandruff reappeared, and CP shampoo was prescribed. Although an initial improvement was observed after 2 weeks, the therapeutic effect diminished after 1 month (Figure 1(a)).

### 2.2. Case 2

**Figure 2:**
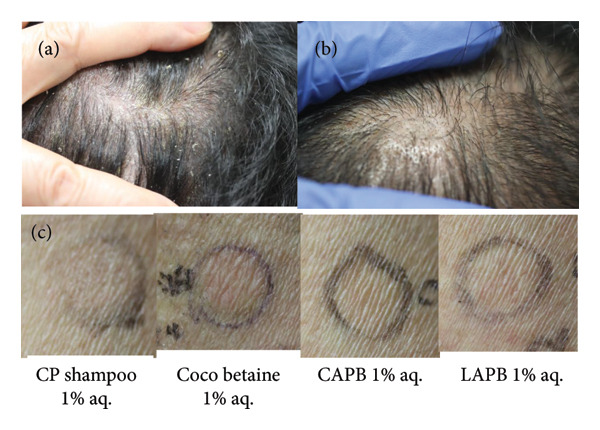
Clinical (a, b) and patch test findings obtained on D3 (c) in Case 2. Clinical findings of Case 2 before (a) and after (b) the use of CP shampoo was discontinued: (a) pronounced erythema with rough scaling and (b) a marked improvement. (c) Positive patch test results for coco betaine 1% aq. and doubtful results for CP shampoo 1% aq., CAPB 1% aq., and LAPB 1% aq. on D3. CP: clobetasol propionate, CAPB: cocamidopropyl betaine, LAPB: lauramidopropyl betaine.

A 52‐year‐old Japanese male developed scalp dermatitis and was prescribed betamethasone butyrate propionate lotion. Despite changing to a less irritating shampoo after a positive patch test of his previous shampoo, his condition did not improve. The subsequent prescription of CP shampoo led to a slight improvement, but his scalp dermatitis worsened after 22 months (Figure 2(a)).

Both patients had a history of severe atopic dermatitis since early childhood and had been treated with 100 mg/day (D) cyclosporine since the ages of 45 and 47, respectively.

Patch testing was performed using CP shampoo, its constituents, CAPB, and LAPB, the latter two being amphoteric surfactants that are frequently found in shampoos. In Case 1, patch tests of dimethylaminopropylamine (DMAPA) and lauramidopropyl dimethylamine (LAPDMA) (an amidoamine [AA]), which may be the real allergens in CAPB [4, 5], were also performed. Finn Chambers (SmartPractice, Phoenix, Arizona) were used as patch test units, with readings taken on D2, D3, and D7, according to the International Contact Dermatitis Research Group criteria [6]. Written informed consent was obtained from the patients. Table 1 and Figures 1(c) and 2(c) present the patch test results for our cases. In Case 1, the patient showed positive reactions to CP shampoo 1% aq., coco betaine 1% aq., CAPB 1% aq., and LAPB 1% aq., but a negative reaction to CP 1% pet., DMAPA 1% aq., and LAPDMA 0.1% aq. In Case 2, the patient exhibited a positive reaction to coco betaine 1% aq., but a doubtful reaction to CP shampoo 1% aq., CAPB 1% aq., and LAPB 1% aq.

**Table 1:** Patch test results of Cases 1 and 2.

	Allergen	Conc./vehicle	Case 1		Case 2	
			D3	D7	D3	D7
Product that caused dermatitis	Clobetasol propionate (CP) shampoo	1% aq.	+	+?	−	+?

Ingredients of the product	Clobetasol propionate	0.05% pet. 1% pet.	− −	− −	− NT	− NT
	Ethanol	60% aq.	+?	−	−	−
	Coco betaine	1% aq.	+	−	+	+
	Sodium laureth sulfate	0.2% aq.	−	−	−	−
	Polyquaternium‐10	0.1% aq.	−	−	−	−
	Citric acid monohydrate	1% aq.	−	−	−	−
	Sodium citrate	1% aq.	−	−	−	−
	Petrolatum	*as is*	−	−	−	−

Amphoteric surfactants	Cocamidopropyl betaine (CAPB) lauramidopropyl betaine (LAPB)	1% aq. 1% aq.	+ +	+ −	+? +?	+? +?

Impurities present in CAPB	Dimethylaminopropylamine (DMAPA) Lauramidopropyl dimethylamine (LAPDMA)	1% aq. 0.1% aq.	− −	− −	NT NT	NT NT

*Note:* Conc., concentration; D, day; +, weak positive reaction; +?, doubtful reaction; −, negative reaction; NT, not tested.

Based on these results, we concluded that the coco betaine in the CP shampoo was the causative allergen in both cases. The patients’ dermatitis markedly improved after they stopped using the CP shampoo (Figures 1(b) and 2(b)).

## 3. Discussion

This is the first report to detail two cases of allergic contact dermatitis caused by CP shampoo, and coco betaine was identified as the causative allergen in both cases. In addition, patch tests of CAPB and LAPB were conducted in both cases, and patch tests of DMAPA and LAPDMA were performed in one case.

**Figure 3:**
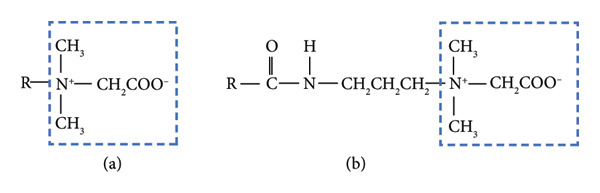
Chemical formulas coco betaine (a) and cocamidopropyl betaine (b). Both surfactants share a core betaine structure, i.e., a 2‐(alkyl dimethyl ammonio)acetate structure, as indicated by the dotted squares. R represents the alkyl groups derived from coconut oil.

Coco betaine and CAPB/LAPB are both amphoteric surfactants derived from coconut oil [7, 8], which have multiple functions in cosmetic products, e.g., they are used as foaming agents, hair‐ and skin‐conditioning agents, antistatic agents, surfactant‐cleansing agents, and viscosity‐increasing agents. Figure 3 shows the chemical formulas of coco betaine and CAPB. LAPB forms when the alkyl group of lauric acid is used as the R‐group in CAPB. According to the Cosmetic Ingredient Review (CIR) Expert Panel, coco betaine, along with the other 10 alkyl betaines, is considered safe as a cosmetic ingredient, based on the concentrations it is currently used at and present practices, as described in their safety assessment [7]. Specifically, when used at concentrations up to 5%, coco betaine was not found to be sensitizing in a nonhuman dermal study. Prior to this, the panel had also reviewed the safety of CAPB and related amidopropyl betaines, such as LAPB, concluding that these ingredients are safe for use in cosmetics when formulated to be nonsensitizing [8].

Despite the CIR’s safety assessment, sensitization to coco betaine and CAPB/LAPB has occurred in a few cases. Table 2 summarizes three previously reported cases [2, 3] and two of our cases of allergic contact dermatitis attributable to coco betaine. The five patients, comprising three males and two females aged 22–52 years, developed dermatitis associated with the use of shampoos in four cases (including two involving CP shampoo) and a beard cleanser in one case. Patch testing with coco betaine yielded uniformly positive reactions (+ to +++), although the concentrations tested varied between 1% and 6%. Patch testing with the respective products demonstrated positive reactions (+ to +++) in four cases, whereas one case (our Case 2) exhibited a doubtful reaction. Patch testing with CAPB was conducted in all cases, resulting in two positive, one doubtful, and two negative reactions. Patch testing of DMAPA and LAPDMA was performed in two cases and one case, respectively, all of which were negative.

**Table 2:** Previously reported cases of allergic contact dermatitis due to coco betaine (including our cases).

	Age, sex	Product that caused dermatitis	Sites of dermatitis	Patch test results (conc., vehicle)			References	
				Product	Coco betaine	Other related chemicals	Authors	Year
1	44 F	Shampoo	Back, palms, scalp	++ (2% aq.) (open test *as is* ++)	+++ (2% aq.)	Parahydroxybenzoic acid esters ++ (5% pet.) CAPB +	Van Haute and Dooms‐Goossens [2]	1983
2	22 F	Shampoo	Face, shoulders, scalp	+++ (2% aq.) (open test *as is* +++)	+++ (2% aq.)	Sodium lauryl ester sulfate ++ (2% aq.) CAPB ‐	Van Haute and Dooms‐Goossens [2]	1983
3	51 M	Beard cleanser	Face, neck	++ (*as is*) ROAT +	++ (6% aq.)	CAPB − (1% aq.) DMAPA − (1% pet.)	Badaoui [3]	2024
4	49 M (our Case 1)	Clobetasol propionate (CP) shampoo	Scalp	+ (1% aq.)	+ (1% aq.)	CAPB + (1% aq.) LAPB + (1% aq.) DMAPA − (1% aq.) LAPDMA − (0.1% aq.)	Iijima et al.	2025
5	52 M (our Case 2)	Clobetasol propionate (CP) shampoo	Scalp	+? (1% aq.)	+ (1% aq.)	CAPB +? (1% aq.) LAPB +? (1% aq.)	Iijima et al.	2025

*Note:* F, female; M, male; aq., aqueous; pet., petrolatum; CAPB, cocamidopropyl betaine; LAPB, lauramidopropyl betaine; DMAPA, dimethylaminopropylamine; LAPDMA, lauramidopropyl dimethylamine; +, weak positive reaction; ++, strong positive reaction; +++, extreme positive reaction; +?, doubtful reaction; −, negative reaction.

Abbreviation: ROAT, repeated open application test.

Doubtful patch test reactions generally do not meet the patch testing criteria for positivity. Recently, such reactions have been analyzed to determine whether they indicate true allergies. Reeder et al. [9] concluded in their study that doubtful reactions to CAPB 1% aq. were less likely to be interpreted as allergic reactions. However, Arora et al. [10] demonstrated that certain doubtful reactions to CAPB were clinically relevant.

Van Haute and Dooms‐Goossens [2] suggested that cross‐reactivity between coco betaine and CAPB may have occurred due to the core betaine structure, i.e., the 2‐(alkyl dimethyl ammonio)acetate structure, shared by these molecules (Figure 3). In Case 1, positive reactions to both CAPB and LAPB in addition to coco betaine were observed, and a shampoo that the patient had previously used contained CAPB, suggesting that he had a preexisting allergy to CAPB. The patient then developed a coco betaine allergy shortly after using CP shampoo, which suggests that the coco betaine allergy occurred through concomitant sensitization rather than cross‐reactivity. In contrast, the patient in Case 2 developed a coco betaine allergy after using CP shampoo for an extended period and exhibited doubtful reactions to both CAPB and LAPB. This suggested that the patient was initially sensitized to coco betaine and subsequently exhibited cross‐reactions to CAPB and LAPB.

In Case 2, the patient showed a negative patch test reaction to CP shampoo on D3 and a doubtful reaction on D7. This may be explained by the presence of superpotent corticosteroids in the formulation, which may have suppressed the patch test response. Forkel et al. [11] recommended late patch test readings on D7 to avoid missed diagnoses, and in a study of 401 patients, 5.9% of corticosteroid reactions first became positive on D7. In our Case 2, the D7 reading was slightly stronger than the D3 reading, but no readings were performed after D7.

Also, the patient in Case 2 was receiving immunosuppressive medication, cyclosporine, for severe atopic dermatitis at the time of the patch testing. An international survey of experts from North America, Europe, Asia, South America, and Oceania reported varying opinions regarding the influence of immunomodulatory therapy on patch test results [12]. According to this survey, 10% of experts believed that cyclosporine has little or no effect, 30% recommended performing patch tests with reduced doses, and 42% advised against testing during immunosuppressive therapy.

It is worth noting that our two patients had severe atopic dermatitis since childhood. There is much debate over whether atopic patients are more susceptible to allergic contact dermatitis than nonatopic patients. According to a recent review article by Simonsen et al. [13], nonatopic patients generally are not at a higher risk of contact sensitization than atopic patients. Hamann et al. [14] concluded that there was no difference in the prevalence of contact allergies between atopic and nonatopic patients. However, Johnson et al. [15] demonstrated that the prevalence of positive patch test reactions to CAPB was significantly higher in children with atopic dermatitis than in those without it (9.3% vs. 2.7%, *p* value = 0.007) in a study of 14 geographically diverse centers in the United States. Also, Lubbes et al. [16] analyzed 1083 Dutch patients under 18 years of age, who were treated at three university hospital allergology units, and CAPB was one of the allergens that frequently produced positive reactions, with such reactions occurring significantly more often in children with atopic dermatitis (17% vs. 9%, *p* value = 0.018).

The patch test results for DMAPA and LAPDMA (AA) in Case 1 and a previously reported case [3] were all negative. These chemicals are suspected to be the true allergens in CAPB. We recently reported that only half of patients allergic to CAPB/LAPB produced positive results for DMAPA and/or LAPDMA in patch testing [17]. Although CAPB and LAPB contain DMAPA and LAPDMA as impurities derived from the manufacturing process, coco betaine, a type of alkyl betaine, does not contain them [7].

In Japan, only 1.4% of shampoos contain coco betaine; however, 64.3% of shampoos contain CAPB and/or LAPB [18]. Therefore, dermatologists should carefully select nonallergenic shampoos for patients with contact allergies to the coco betaine in CP shampoo, because some patients may also be allergic to CAPB/LAPB. In addition, in cases of recalcitrant scalp dermatitis, it is necessary to consider the possibility that the topical medication being used may be causing the condition and to be aware that the “active” ingredient is not always the culprit.

## Conflicts of Interest

The authors declare no conflicts of interest.

## Funding

No funding was received for this study.

## Data Availability

The data that support the findings of this study are available on request from the corresponding author. The data are not publicly available due to privacy or ethical restrictions.
